# Benzothiazole derivatives target oligoadenylate synthetase and enhance RSV vaccine efficacy as adjuvants

**DOI:** 10.1016/j.bioactmat.2026.07.049

**Published:** 2026-08-04

**Authors:** Xuanli Jin, Zhihui Zhang, Hong Liu, Yunzhi Tan, Peiying Luo, Zhenfu Wen, Haolin Chen, Lixin Liu, Yongming Chen

**Affiliations:** a School of Materials Science and Engineering, Key Laboratory for Polymeric Composite and Functional Materials of Ministry of Education, Sun Yat-sen University, Guangzhou, 510275, China; b School of Medical Technology, Xuzhou Medical University, Xuzhou, 221004, China; c Guangxi Academy of Medical Sciences, The People's Hospital of Guangxi Zhuang Autonomous Region, Nanning, Guangxi, 530021, China; d College of Chemistry and Molecular Science, Henan University, Kaifeng, Henan, 475004, China; e Translational Medical Center of Huaihe Hospital, Henan University, Kaifeng, 475004, China; f State Key Laboratory of Antiviral Drugs, Henan University, Zhengzhou, 450046, China

**Keywords:** Benzothiazole derivatives, OAS agonist, Adjuvant, RSV pre-F

## Abstract

2′,5’ oligoadenylate (2-5A) activates RNase L to degrade intracellular RNA and inhibit viral proliferation. The resulting nucleotide fragments bind RIG-I-like receptors to initiate type I interferon (IFN-I) signaling, enhancing maturation of antigen processing cells to trigger adaptive immunity. Moreover, 2-5A is an immune mediator to initiate neighboring cells for antiviral immunity. However, due to its short half-life in biological systems hinders its application in vaccine. Herein, we present a series of benzothiazole derivatives with modification at 2, 3, and 6 positions, that exhibit enhancing antigen immunogenicity. Among them, 3-benzyl-6-hydroxy-2-methylbenzo[d]thiazol-3-ium bromide and 3-benzyl-6-methoxy-2-methylbenzo[d]thiazol-3-ium bromide, named as CG and DG, significantly promoted dendritic cell maturation and elevated IgG titers when combination with model antigen OVA. By transcriptome sequencing analysis and knock out experiments, they activated 2-5A synthetase (OAS) to increase 2-5A production, and further was corroborated by *in vitro* OAS activity assay. When combined with pre-F antigen from respiratory syncytial virus (RSV), CG and DG significantly increased populations of memory B and T cells and enhanced neutralizing antibody titers. Thus, this study developed two small non-nucleotide agonists, CG and DG, that may active the OAS-2-5A-RIG-I innate sensing axis. Their stable chemical structure and clear molecular target OAS render them as promising adjuvant for technology translation.

## Highlights

• CG and DG act as non-nucleotide OAS agonists that specifically activate the OAS-2-5A-RIG-I innate immune signaling axis.
• CG and DG effectively promote dendritic cell maturation and elevate antigen-specific IgG responses with favorable biosafety.
• CG and DG significantly augment memory B/T cell populations and neutralizing antibody titers in RSV pre-F vaccines with translational potential.

## Graphical abstract

**Figure:**
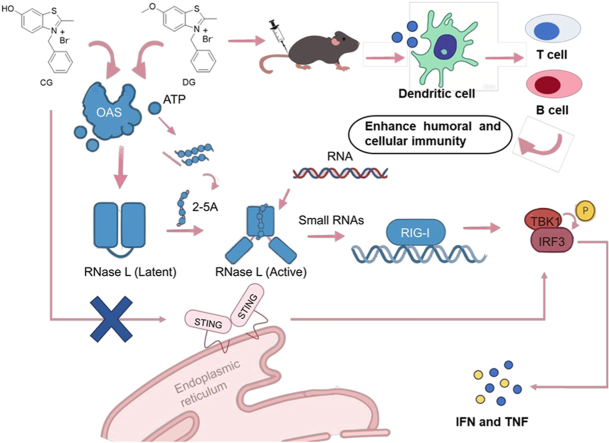

## 1 Introduction

**Scheme 1:**
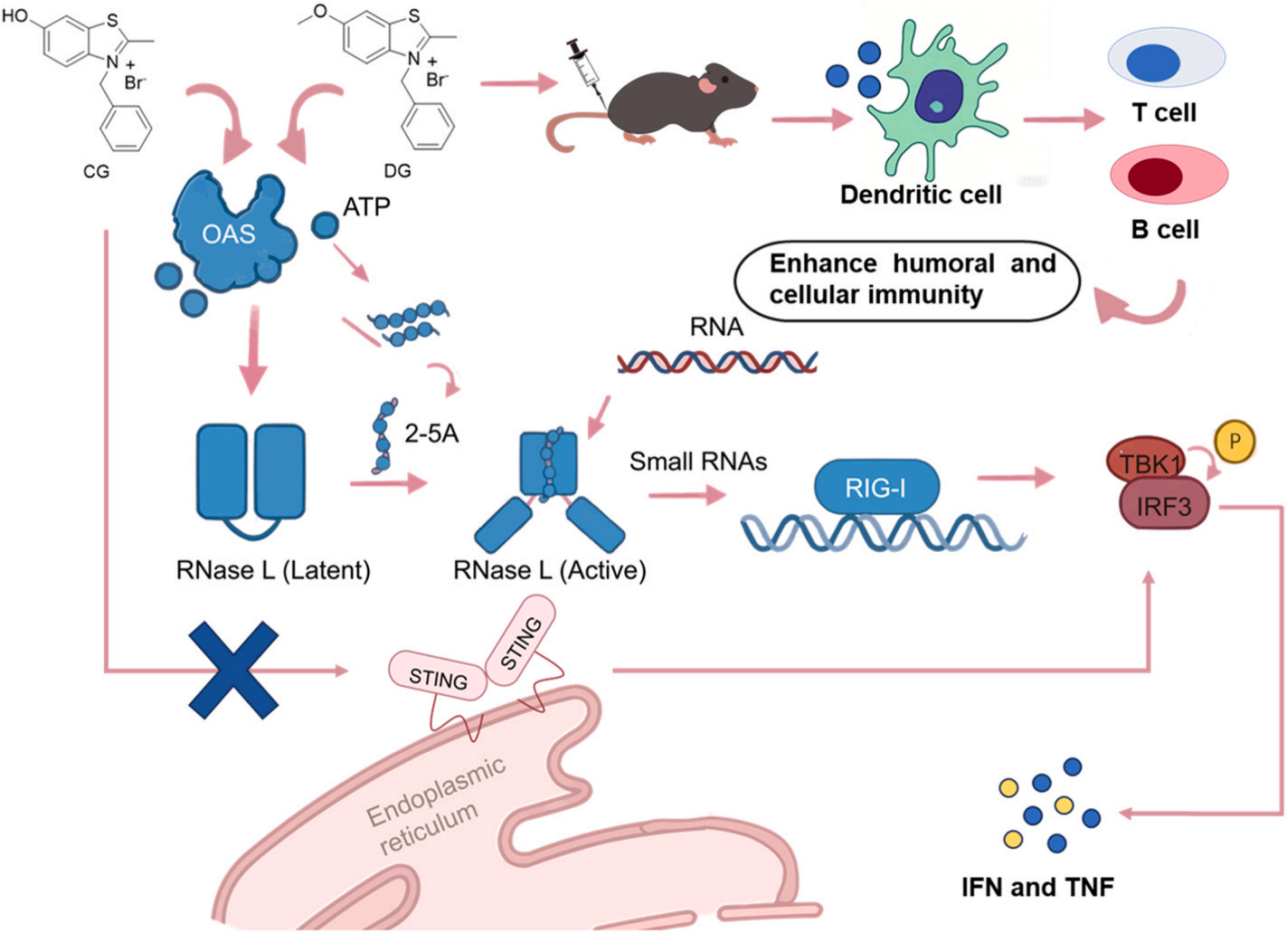
Benzothiazole derivatives as non-nucleotide agonists targeting the 2′,5′ oligoadenylate synthetase (OAS) pathway for vaccine application.

The molecules of pathogen-associated molecular patterns (PAMPs) have been widely developed as high-efficiency adjuvants to activate innate immunity and enhance vaccine's efficacy [1,2]. For instance, Toll-like receptors (TLRs), a class of transmembrane pattern-recognition receptors (PRRs), can recognize distinct PAMPs (e.g., TLR4 recognizes lipopolysaccharide, TLR3 recognizes double-stranded RNA, and TLR9 recognizes CpG DNA) [3,4].Their agonists have been widely investigated in preclinical and clinical studies, demonstrating significant efficacy to enhance vaccine efficacy by activating innate immune responses and bridging adaptive immunity. Besides these extracellular and transmembrane PRRs, cyclic GMP-AMP synthase (cGAS), the intracellular abnormal double-stranded DNA (dsDNA) sensor, once activated, it produces 2′,3′-cGAMP to activate stimulator of interferon genes (STING), induce the secretion of interferons and proinflammatory cytokines, and enhance antigen presentation [5,6]. More importantly, 2′,3′-cGAMP can be the second messenger molecule being transmitted to neighboring cells to initiate the STING pathway to amplify immune activation signals, further expanding the scope and intensity of innate immune responses [7]. Thereby, adjuvants targeting cGAS-STING activation, including nucleotide-based, non-nucleotide small-molecule, and metal ion-based types, have been extensive explored [8] (see Scheme 1)

2′,5′ oligoadenylate synthetase (OAS) is homologous to cGAS, belonging to the nucleoside transferase superfamily, and can be activated by viral double-stranded RNAs (dsRNAs) [9]. Once being activated, OAS catalyzes ATP polymerization to form 2′,5′-oligoadenylate (2-5A) molecules, which then activates RNase L to degrade viral RNAs, whose fragments transactivate RIG-I, amplifying interferon responses and enhancing antigen presentation. Like 2′,3′-cGAMP, 2-5A is also an immune-transmitter and immunostimulant of OAS-2-5A-RIG-I, having a great potential in cancer immunotherapy and antiviral infection and has the potential to be used as a vaccine adjuvant [[10], [11], [12], [13]].However, due to 2-5A was easily degraded in biological systems hinders its application in vaccines.

Herein, we synthesized a series of benzothiazole derivatives and explored their performance as adjuvants. After screening with THP-1 cells, 3-benzyl-6-hydroxy-2-methylbenzo[d]thiazol-3-ium bromide and 3-benzyl-6-methoxy-2-methylbenzo[d]thiazol-3-ium bromide, named as CG and DG, potently activated TBK1, inducing strong expression of type I interferon and TNF-α. When combined with model antigen OVA, they effectively drove DC maturation and enhanced the IgG production. Subsequently, taking pre-fusion F protein (pre-F) from respiratory syncytial virus (RSV) as antigen, the efficacy of the adjuvanted vaccines has been evaluated. When co-administered subcutaneously with pre-F antigen, CG and DG significantly increased the levels of antigen-specific neutralizing antibodies and memory T/B cells without inducing significant toxicity. After transcriptome sequencing (RNA-seq) analysis and knock out experiment, the target genes of CG and DG were identified as OAS, and further was corroborated by *in vitro* OAS activity assay. They can activate OAS enzymatic activity to generate 2-5A, subsequently, initiating the downstream RIG-I-like receptors to initiate type I interferon (IFN-I) signaling. In contrast to 2-5A, benzothiazole scaffolds are chemically stable and we disclosed their significant performance in activating OAS signaling pathway. Thus, this work presented the discovery of unique non-nucleic small molecules, demonstrated their targeting of the OAS pathway as agonist response, and thereby revealing their potential as vaccine adjuvants against pathogen infections.

## 2 Results

### 2.1 Synthesis and characterization of benzothiazole derivatives

**Fig. 1:**
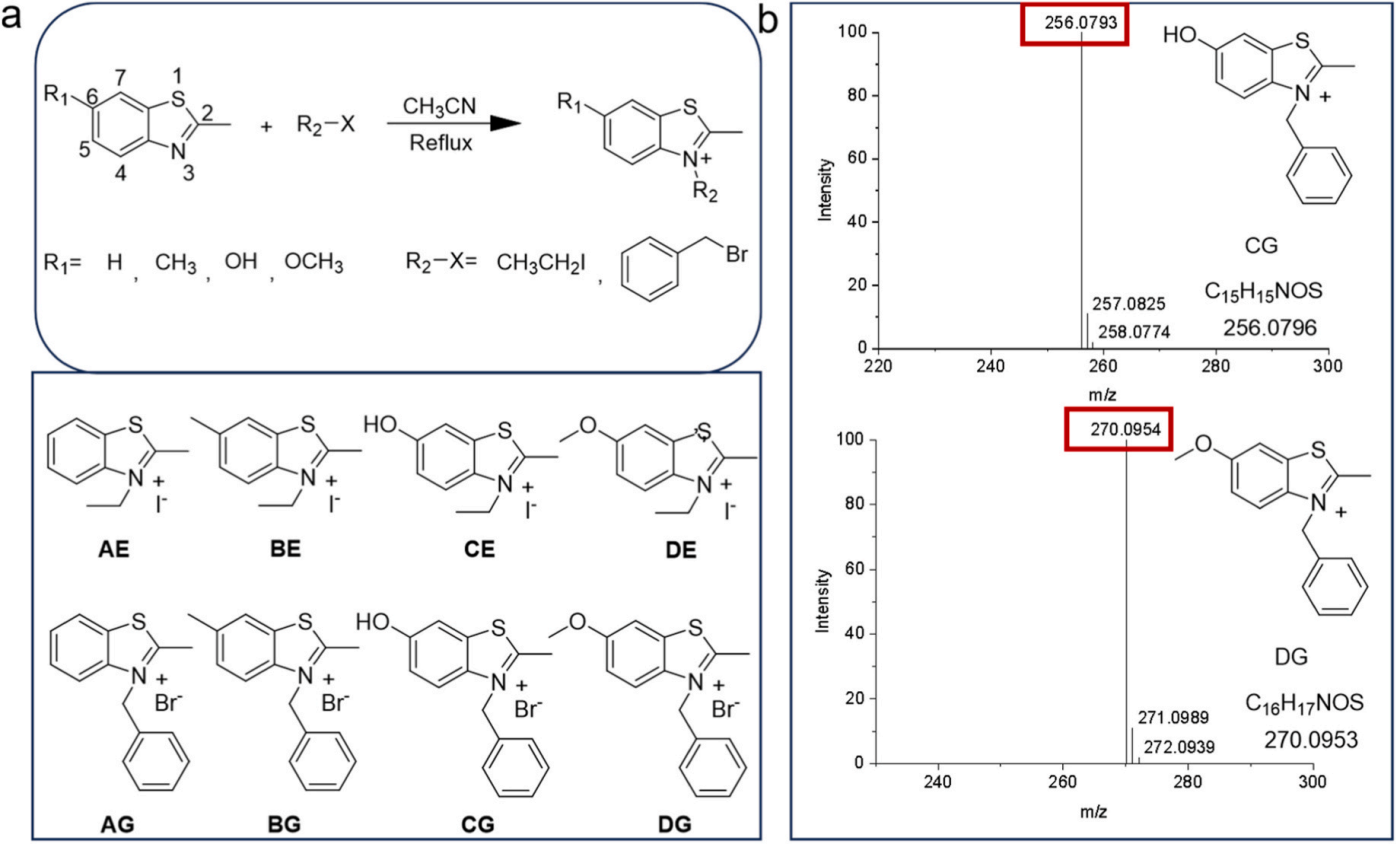
**Synthesis and structural verification of benzothiazole derivatives.** (a) Synthetic route for benzothiazole derivatives. By introducing different substituents at R_1_ sites (hydrogen, methyl, hydroxyl and methoxy) and R_2_ sites (ethyl, benzyl), a small library of molecules containing eight members (compounds AE, BE, CE, DE, AG, BG, CG, DG) was constructed. (b) High-resolution mass spectrometry (HRMS) characterization of representative benzothiazole derivatives.

Benzothiazole (BZT) is a planar bicyclic skeleton formed by the fusion of a benzene ring with a thiazole ring containing a sulfur atom at position 1 and a nitrogen atom at position 3 (Fig. 1a). The planar structure of this fused heterocycle enables key intermolecular interaction such as π-π stacking with biological targets including nucleic acids and proteins. The sulfur and nitrogen heteroatoms not only markedly enhance the capacity of BZT derivatives to form noncovalent interactions with key amino acid residues in the binding sites of proteins, but also strengthen interactions with amino acid residues in protein binding sites and planar pockets of nucleic acids or proteins, thereby improving binding affinity and specificity [14]. In addition, the unique electronic and steric properties of the skeleton allow BZT to interact efficiently with a variety of biological targets [15,16]. Existing studies have shown that the main modification sites of benzothiazole-based drugs are concentrated at the C2, N3 and C6 positions (Fig. 1a). Structure-activity relationship studies have confirmed that the introduction of electron-withdrawing groups at the C2, C5 and C6 positions can significantly improve the biological activity of the compounds, while the introduction of hydrophilic groups at the N3 position can effectively improve aqueous solubility [17,18]. Although a number of benzothiazole-based drugs have been approved for application, the development of many candidate drugs has been terminated due to research and development setbacks, among which compounds modified at C5 have been associated with increased toxicity [19,20]. In contrast, the benzothiazole derivatives modified at the C2, N3 and C6 positions can be incorporated with various pharmacophores to form multi-functional drugs, and a number of relevant candidate molecules are currently in clinical or preclinical development stages [21,22]. This characteristic highlights the sustained research value of the benzothiazole skeleton in meeting the diverse clinical medical needs. Based on the above research background, the C2, N3 and C6 positions were selected as the main modification sites of benzothiazole in this study. To simplify the synthetic process and reduce the potential toxicity of the compounds, short-chain groups were used for subsequent structural modification.

We designed and synthesized a series of derivatives with different substituents at the R_1_ and R_2_ sites based on the 2-methyl-benzothiazole core (Fig. 1a). In terms of design strategy, the substituents with different polarities and electronic properties, substituents including hydrogen, methyl, hydroxyl, and methoxy were introduced at the C6, R1 site, to modulate the interaction between the molecules and potential target proteins; while ethyl or benzyl groups were introduced at the R_2_ site by quaternarization of nitrogen with alkyl halides to evaluate the effects of hydrophobicity and steric effect on immunomodulatory activity. The compound with a hydrogen at the R_1_ position and an ethyl group at the R_2_ position was identified as 3-ethyl-2-methylbenzo[d]thiazol-3-ium iodide, designated as AE. Following the same nomenclature principle, the remaining seven benzothiazole derivatives were named BE, CE, DE, AG, BG, CG, and DG based on their different substituents (Fig. 1a).

The structures of all compounds were characterized by ^1^H NMR, elemental analysis, and high-resolution mass spectrometry (HRMS), and the experimental data were highly consistent with the theoretical structures (Fig. 1b, Figs. S1–S14 and Table S1). For example, the high-resolution mass spectrometry of the representative compound CG showed [M]^+^ *m*/*z* = 256.0793, which is in high agreement with the theoretical value of 256.0796. Elemental analysis results showed that the C, H, N, and S content of each compound deviated from the theoretical value by less than 0.4%, confirming high purity of the target molecules (Fig. 1b). It is worth noting that all compounds were synthesized in one step *via* a quaternization reaction using commercially available benzothiazole as the starting material. The reaction conditions were mild and the yields were high, demonstrating a low cost for potential application. These results indicate that this study obtained a group of benzothiazole derivatives with well-defined structures and reliable purity, providing a solid chemical basis for subsequent research on immune function and mechanisms.

### 2.2 CG and DG serve as adjuvants to enhance model antigen OVA immunogenicity

**Fig. 2:**
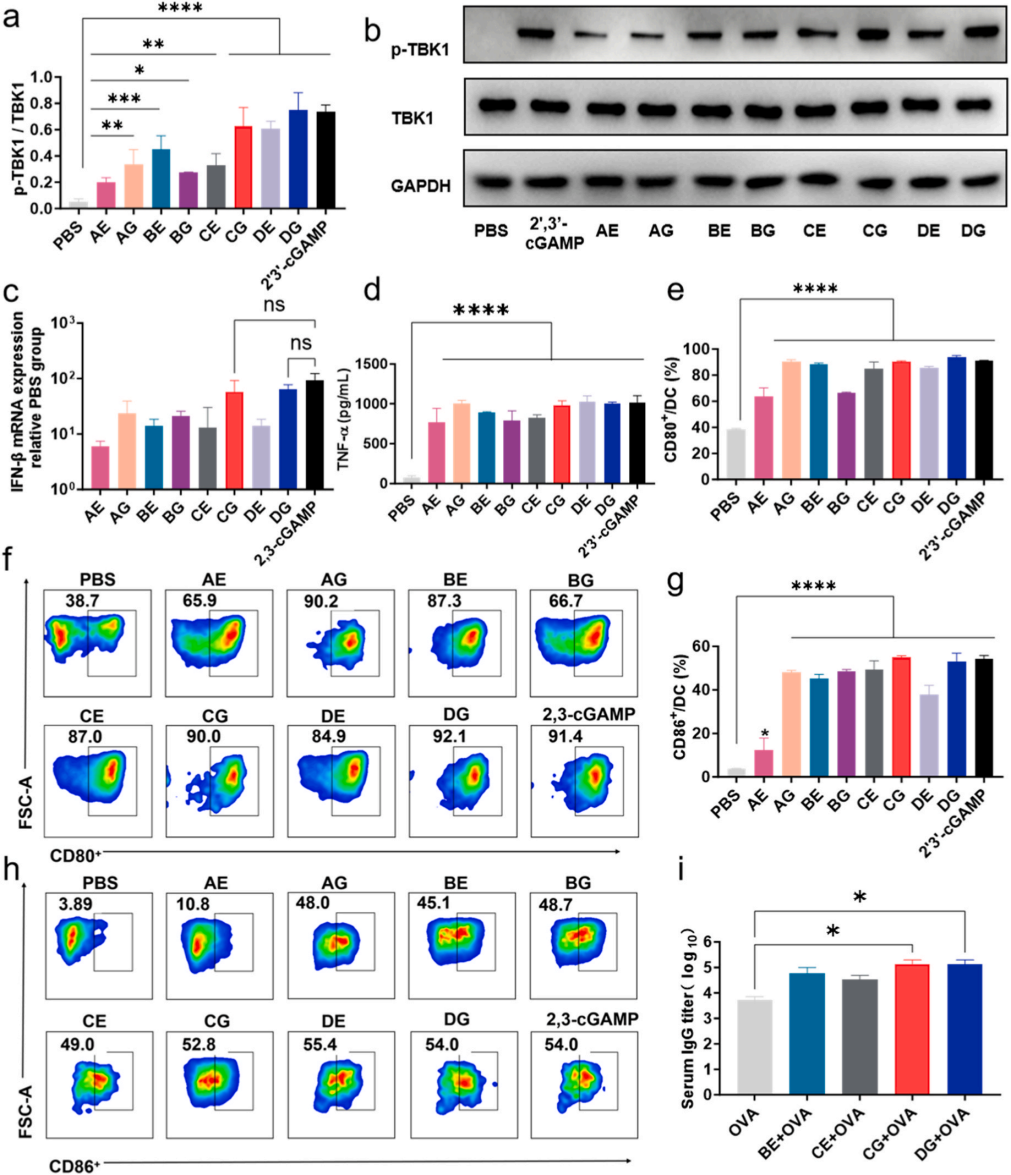
**CG and DG serve as adjuvant to enhance model antigen OVA immunogenicity.** (a-b) Western blot analysis of relative p-TBK1 signal in THP-1 cell treated with PBS, 50 μg/mL of 2′3′-cGAMP, or 50 μg/mL of benzothiazole derivatives for 24 h, normalized to total TBK1 and compared to the 2′3′-cGAMP positive control. (c) RT-qPCR analysis of relative IFN-β mRNA expression levels (PBS group as control). (d) ELISA detection of TNF-α secretion levels. Representative flow cytometry (e) and quantitative analysis (f) of CD80^+^ cells after stimulation with 50 μg/mL of each compound for 24 h. Representative flow cytometry (g) and quantitative analysis (h) of CD86^+^ cells after stimulation with 50 μg/mL of each compound for 24 h. (i) OVA-specific IgG antibody titer in serum of C57BL/6 mice after immunization. C57BL/6 mice were subcutaneously immunized on days 0 and 14 with 10 μg of OVA antigen alone or mixed with 10 μg of designated compounds (BE, CE, CG, DG). Data are represented as mean ± SD (n = 3). *p < 0.05, **p < 0.01, ***p < 0.001, ****p < 0.0001, ns: no statistically significant difference.

Next, the innate immune-activating capability of eight benzothiazole derivatives was evaluated. Firstly, Western blot results showed that all eight benzothiazole derivatives significantly induced phosphorylation of TBK1 kinase. We assessed the phosphorylation of TBK1 (p-TBK1 relative to total TBK1) following treatment with various benzothiazole derivatives, observing that most compounds significantly enhanced TBK1 activation compared to the PBS control. The results revealed that treatment with the tested compounds significantly increased p-TBK1 levels approximately 2–7-fold compared with PBS control, CG, DE, and DG showing strong induction effects, comparable to the activity of the positive control 2′3′-cGAMP (Fig. 2a and b) [23,24].

As RIG-I functions as an upstream signaling adaptor of TBK1, we further quantified the RIG-I induction capacity of the eight candidate compounds [23]. The results demonstrated that all tested compounds significantly upregulated RIG-I expression by approximately 2-3 folds relative to the PBS group. Among them, CE, CG, and DG exhibited potent stimulatory activity, with their induction efficacy comparable to that of the positive control poly I:C (Fig. S15). Collectively, these findings confirm that the candidate compounds efficiently trigger innate immune responses by initiating the RIG-I-TBK1 signaling cascade to modulate downstream immune processes.

Since IFNs are key indicators of innate immune activation, we examined the ability of the compounds to induce IFN-β production in immune cells. After THP-1 cells were treated with 50 μg/mL of each compound for 24 h, the RT-qPCR analysis of IFN-β mRNA expression levels showed that all compounds significantly upregulated IFN-β compared with the PBS control group. Among the compounds, CG and DG showed the most significant activation effects, reaching 56.5-fold and 63.8-fold higher than those of the control groups, respectively. The effects of CG and DG was not statistically significantly different from the positive control 2′,3′-cGAMP (Fig. 2c). Notably, the G-series compounds (AG, BG, CG, DG) with a benzyl group at the N3 site generally exhibited stronger IFN-β induction ability than the E-series compounds (AE, BE, CE, DE) with an ethyl group at the N3 site, suggesting that larger hydrophobic aryl substitution may be more conducive to enhance immune activation. In parallel, we included two BZT core derivatives CM (2-methyl-1,3-benzothiazol-6-ol) and DM (6-methoxy-2-methylbenzothiazole) as controls to exclude intrinsic adjuvant effects of the original benzothiazole structure. CG and DG exhibited robust activation at 23.8-fold and 29.6-fold versus the parent BZT structure, while CM and DM barely induced any response, with expression levels comparable to the PBS blank control. This observation demonstrates that the bare BZT backbone possesses no independent adjuvant activity (Fig. S16).

An ELISA assay was performed to confirm above results, which revealed that compound treatment significantly increased the secretion level of the pro-inflammatory cytokine TNF-α (approximately 4-6 times), with DE, CG and DG showing the strongest induction effects (Fig. 2d). Collectively, these results indicate that benzothiazole derivatives can effectively induce the production of type I interferon and pro-inflammatory factors, activating downstream effects of innate immunity.

As dendritic cells (DC) are key antigen-presenting cells bridging innate and adaptive immunity, their maturation status, expression of co-stimulatory molecules CD80/CD86, directly determines the strength of adaptive immunity [[25], [26], [27]]. Using mouse bone marrow-derived dendritic cells (BMDCs) as a model, we further evaluated the effects of benzothiazole derivatives on DC maturation. Flow cytometric analysis of BMDCs treated with compounds at 50 μg/mL for 24 h confirmed obvious upregulation of co-stimulatory molecules CD80 and CD86 (Fig. 2e–h, Fig. S17). All tested compounds increased the frequency of CD80^+^ cells by 1.5-2.5-fold relative to the PBS group, while CD86^+^ cell proportion was increased to 10-15-fold of the control level. Specifically, both CG and DG exhibited comparable induction capacity towards these two markers with positive control 2′,3′-cGAMP. This indicates that these benzothiazole derivatives can effectively promote DC maturation and enhance their antigen-presenting potential.

Based on *in vitro* immune activation efficiency, we selected four representative compounds, BE, CE, CG, and DG, for preliminary *in vivo* adjuvant effect evaluation. Each compound was mixed with the model antigen ovalbumin (OVA) at a 1:1 (w/w) ratio, and C57BL/6 mice were immunized subcutaneously twice on day 0 and day 14. Serum was collected on day 21 to detect OVA-specific IgG antibody titers. ELISA results showed that CG and DG significantly increased antigen-specific IgG titers by approximately 25.6-fold compared to the OVA-alone immunization group, while BE and CE did not show significant enhancement (Fig. 2i).

In summary, the benzothiazole derivatives, especially CG and DG, can activate TBK1 signaling, thereby inducing strong expression of type I interferon and TNF-α, and effectively driving DC maturation. This *in vitro* immune activation translated into a robust antigen-specific response *in vivo*, demonstrating its promising potential as a novel small-molecule vaccine adjuvant.

### 2.3 Transcriptomic profiling reveals distinct innate immune programs induced by CG and DG

**Fig. 3:**
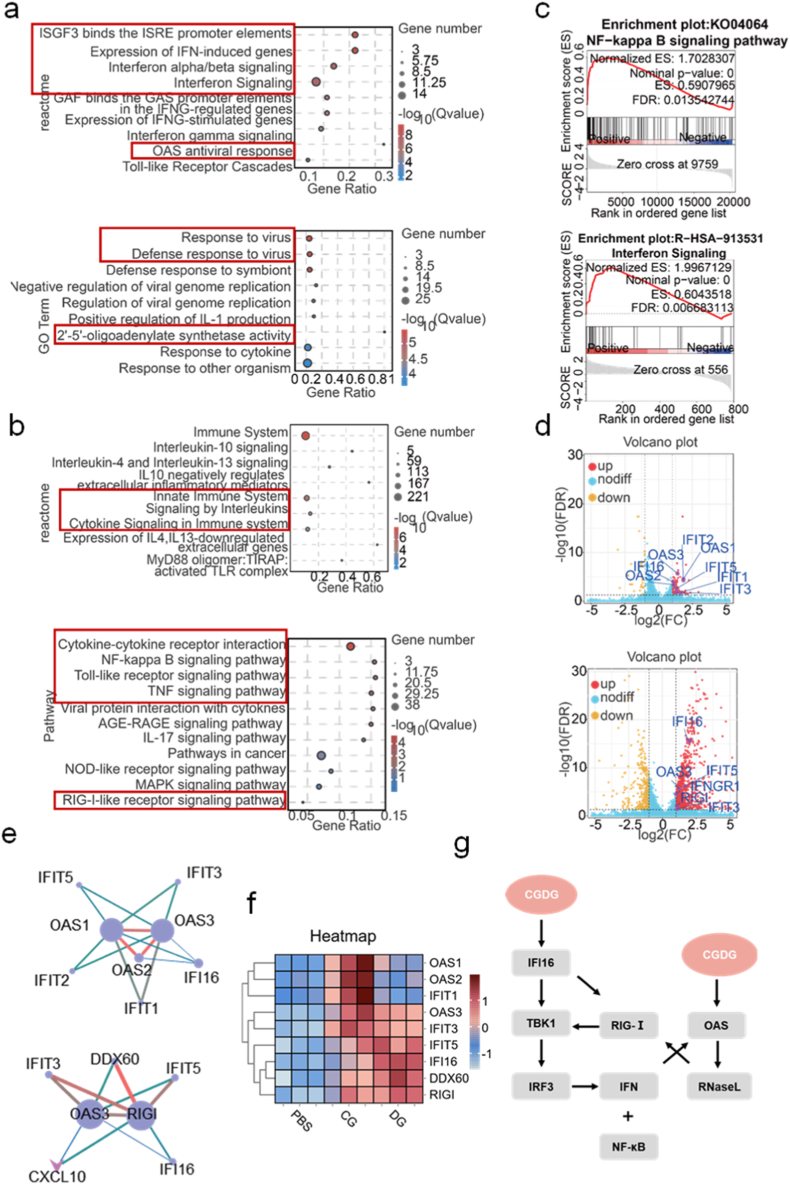
**CG and DG induce distinct antiviral and inflammatory transcriptional programs.** (a) Differential gene pathway enrichment analysis after CG treatment of THP-1 cells. (b) Differential gene pathway enrichment analysis after DG treatment of THP-1 cells. (c) Gene set enrichment analysis plots. Top: GSEA plot of the gene set related to “interferon response” in the CG-treated group; Bottom: GSEA plot of the gene set related to “NF-κB signaling pathway” in the DG-treated group. ES, enrichment score; NES, normalized enrichment score; FDR q, false discovery rate q-value. (d) Volcano plot of differentially expressed genes comparing CG- (top) and DG-treated (bottom) groups with untreated controls (Padj <0.01). Red dots represent significantly upregulated genes. Some key genes have been marked. (e) Protein-protein interaction (PPI) network analysis of key differentially expressed genes in the CG (top) and DG (bottom) treatment groups. (f) Heatmap of expression levels of key immune-related genes in the CG and DG treatment groups relative to the untreated group. (g) Schematic diagram of the mechanism by which CG and DG activate innate immune signaling pathways. By engaging targets such as OAS, these compounds initiate an interferon-driven antiviral program and an NF-κB-mediated inflammatory program, forming a synergistic bimodal immune response.

Based on the above results, CG and DG can efficiently activate TBK1 signaling, subsequently increasing IFN-β and TNF-α expression. To elucidate the molecular mechanisms of action of benzothiazole compounds CG and DG as immune adjuvants, we performed transcriptome sequencing (RNA-seq) analysis on THP-1 cells treated with 50 μg/mL CG or DG for 24 h, with untreated THP-1 cells serving as the control group. Pathway enrichment results based on differentially expressed genes (DEGs) showed that both CG and DG significantly reshaped the innate immune transcriptional landscape (Fig. 3a and b). For the CG-treated group, Reactome and Gene Ontology (GO) enrichment analyses consistently pointed to significant activation of interferon-related innate immune pathways, with highly enriched and significantly upregulated pathways related to “interferon signaling”, “defense response to virus”, and 2′-5′-oligoadenylate synthetase (OAS)-associated antiviral responses. Notably, OAS emerged as a key overlapping component between “antiviral defense response” and “interferon signaling”, suggesting that CG may induce antiviral transcriptional programs through the classic interferon-OAS axis (Fig. 3a) [28]. Conversely, DG treatment was significantly enriched in inflammation-related pathways such as the NF-κB signaling pathway and interleukin signaling (Fig. 3b). These pathways are widely considered to be core downstream signaling modules following the recognition of PAMPs by the innate immune system, suggesting a stronger association with inflammatory innate immune signaling [29]. Gene set enrichment analysis (GSEA) further confirmed that CG treatment specifically activated the interferon response program (ES = 0.60, FDR q = 6.62 × 10^−3^), while DG treatment was strongly enriched in the NF-κB signaling pathway (ES = 0.59, FDR q = 1.35 × 10^−2^) (Fig. 3c). Notably, the DEGs highlighted in the volcano plot were highly enriched in these key pathways. In the CG-treated group, interferon effector gene clusters, interferon-induced protein with tetratricopeptide repeats (IFIT1, IFIT2, IFIT3, IFIT5), OAS, and interferon-inducible protein 16 (IFI16) were significantly upregulated. In the DG-treated group, antiviral and inflammation-related genes such as RIG-I, IFI16, OAS, and IFNGR1 were significantly upregulated, suggesting a stronger tendency to induce viral RNA recognition and inflammatory signaling (Fig. 3d) [30,31].

To further elucidate the immune signaling network structure induced by CG and DG, we constructed a protein-protein interaction (PPI) network of key differentially expressed genes based on the STRING database (Fig. 3e). In the CG group, OAS had the highest gene abundance and formed a highly interactive network with members of the IFIT family. In the DG-treated group, RIG-I was identified as the central hub in the network, forming strong interactions with IFIT, IFI16, and helicase DDX60 [[32], [33], [34]]. Notably, DDX60 is considered a key amplifier of RIG-I-dependent antiviral signaling, and its significant enrichment further supports the strong activation of RIG-I-related pathways by DG [33]. Heatmap analysis of differentially expressed genes further validated these conclusions (Fig. 3f). Although the upstream regulators enriched for interferon-stimulated genes (ISGs) differed between the CG and DG groups, both treatments significantly upregulated ISGs. Based on these findings, an association graph was constructed (Fig. 3g). Specifically, CG and DG may engage OAS-associated antiviral signaling and other innate immune sensing pathways, including IFI16-related responses, which subsequently activate TBK1 and IRF3, thereby inducing interferon transcription and the production of inflammatory factors.

### 2.4 CG and DG activate innate immunity through targeting OAS

**Fig. 4:**
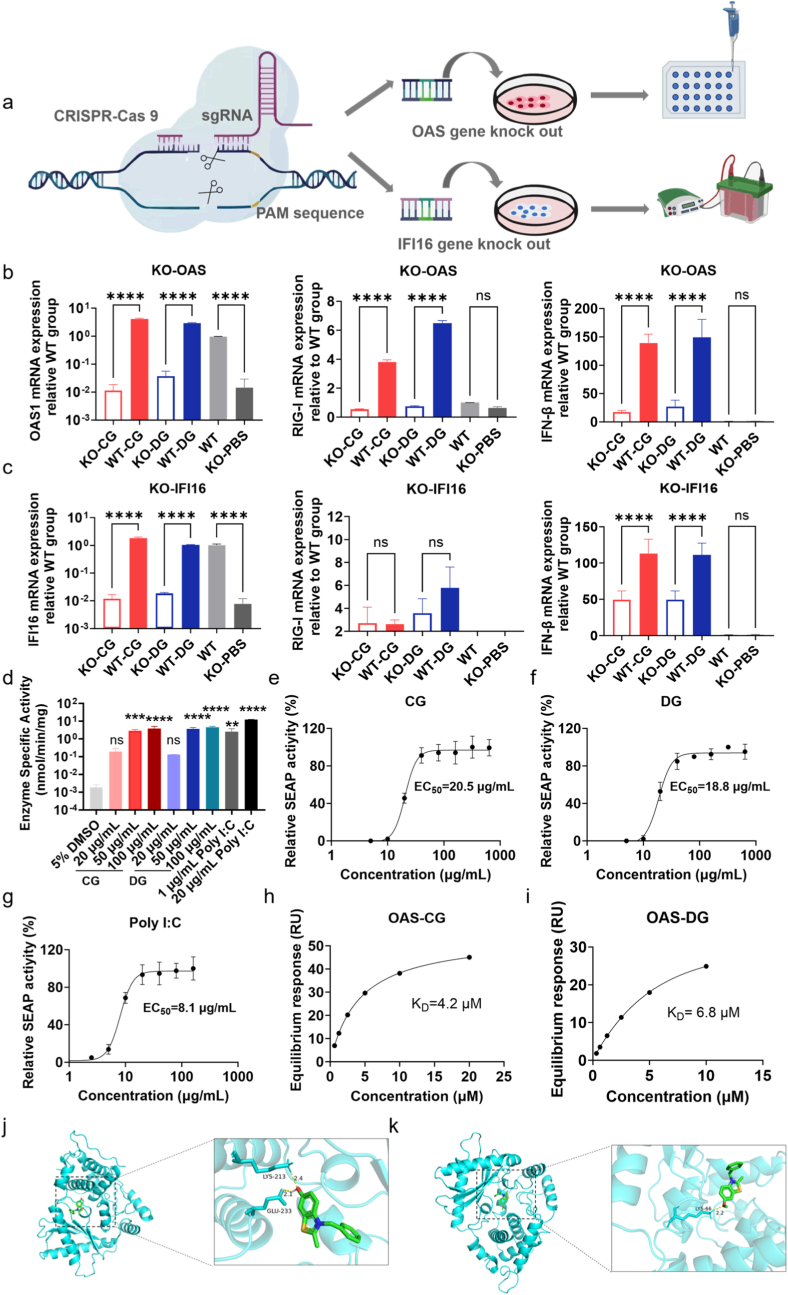
**OAS mediates CG- and DG-induced activation of innate immunity.** (a) Schematic diagram of IFI16 and OAS gene knockout by CRISPR/Cas9 and validation experiments. (b) Relative mRNA expression levels of OAS, RIG-I and IFN-β in WT and KO-OAS THP-1 cells after CG or DG treatment. All mRNA expression values were normalized to the corresponding WT-PBS group, and the vertical axis represents the fold change relative to WT-PBS control. (c) Relative mRNA expression levels of IFI16, RIG-I and IFN-β in WT and KO-IFI16 THP-1 cells after CG and DG treatment. (d) Activation of OAS enzymatic activity by CG and DG at indicated concentrations. Dose-response profiles of benzothiazole derivatives in RAW-BLUE cells treated with CG (e), DG (f) (5, 10, 20, 40, 80, 160, 320, 640 μg/mL), or the positive control Poly I:C (g) (1, 2.5, 5, 10, 20, 40, 80, 160, μg/mL) for 24 h, with NF-κB activation measured *via* SEAP activity and EC_50_ values determined by non-linear regression. Real-time binding sensorgrams of gradient concentrations of CG (h) and DG (i) flowed over immobilized OAS protein, with the dissociation constants (Kd) of 4.2 μM and 6.8 μM obtained by nonlinear regression fitting, respectively. Optimal binding conformation of CG (j) and DG (k) to OAS predicted by AutoDock Vina molecular docking. Data are represented as mean ± SD (n = 3); one-way ANOVA was used for statistical analysis. *p < 0.05, **p < 0.01, ***p < 0.001, ****p < 0.0001, ns indicates no statistically significant difference.

To clarify the direct targets of CG and DG in innate immune activation, we constructed OAS knockout (KO-OAS) and IFI16 knockout (KO-IFI16) THP-1 cell lines and systematically evaluated the expression changes of key immune genes and the activation status of downstream signaling. After confirming that OAS expression was efficiently depleted in KO-OAS cells (Fig. S18), CG and DG were added to KO-OAS cells, using WT cells as the control. As expected, OAS expression was readily detected in WT cells, while it was absent in KO-OAS cells (Fig. 4b). Simultaneously, in KO-OAS cells, the induced expression of downstream signaling molecules RIG-I and IFN-β was fundamentally weakened. In WT cells, CG treatment upregulated RIG-I and IFN-β expression by approximately 7.0-fold and 7.9-fold, respectively, while DG treatment upregulated them by 8.7-fold and 5.5-fold, respectively. However, upon CG or DG stimulation, OAS KO cells exhibited only residual upregulation of RIG-I and IFN-β (≈1.2-1.4-fold), confirming that robust induction is largely OAS-dependent (Fig. 4b). In parallel, we conducted parallel experiments in KO-IFI16 cells. Although IFI16 knockout was confirmed, the ability of CG and DG to induce RIG-I expression was not significantly inhibited, and the upregulation of RIG-I induced by CG or DG treatment was comparable to that in WT cells (Fig. 4c). Similarly, CG- and DG-induced IFN-β mRNA expression was substantially maintained in KO-IFI16 cells. Although the induction level was modestly reduced compared with WT cells, IFN-β transcripts remained significantly elevated relative to PBS controls, indicating that IFN-β induction by CG/DG is largely independent of IFI16. This indicates that CG and DG target OAS to initiate the innate immunity *via* OAS-2-5A-RIG-I pathway.

To further validate activation of the downstream signaling cascade of CG and DG, we examined the phosphorylation status of TBK1, a key downstream kinase in the RIG-I pathway. Western blot analysis showed that in KO-OAS cells, neither CG nor DG effectively induced TBK1 phosphorylation, which is consistent with the result of blocked RIG-I/IFN-β gene induction. On the contrary, in KO-IFI16 cells, TBK1 phosphorylation did not decrease when compared with those in WT cells in three replicate experiments (Fig. S19). This result was further validated by an *in vitro* OAS activity assay with AMP-Glo™ Detection System, which is a homogeneous, luminescence-based assay from Promega used to measure AMP concentration (Fig. 4d). In this system, when OAS was activated by CG and DG, it catalyzed ATP to synthesize 2-5A. Subsequently, the phosphodiesterase 12 (PDE12) hydrolyzes 2-5A to generate adenosine monophosphate (AMP), allowing AMP production to serve as an indirect readout of OAS catalytic activity. When applied to the system, both CG and DG exhibited dose-dependent OAS activity, compared with the 5% DMSO control group. At a concentration of 50 μg/mL of CG and DG, they showed comparable activity to Poly I:C, which is a known OAS agonist used as a positive control.

RNase L and RIG-I serve as critical signaling components of the OAS-RIG-I pathway. To further verify the activated pathway, we treated CG- and DG-stimulated cells with RNase L-IN-1 (RNase L inhibitor) and RIG012 (RIG-I inhibitor), and quantified downstream IFN-β expression *via* q-PCR. Q-PCR results revealed that IFN-β induction in inhibitor groups was reduced to 40-50 folds relative to the CG and DG single treatment groups, confirming that RNase L and RIG-I activation are indispensable for pathway transduction (Fig. S20).

To quantitatively evaluate the immunostimulatory potency of CG and DG and define their dose-response relationships, we performed concentration-gradient stimulation assays in RAW-BLUE cells, which stably express an NF-κB-responsive secreted embryonic alkaline phosphatase (SEAP) reporter for quantitative readout of innate immune activation. Cells were treated with serial concentrations of the benzothiazole derivatives CG or DG (5, 10, 20, 40, 80, 160, 320, 640 μg/mL), or the positive control Poly I:C (1, 2.5, 5, 10, 20, 40, 80, 160, μg/mL) for 24 h, and SEAP activity in culture supernatants was then measured. All compounds exhibited clear concentration-dependent NF-κB activation, with sigmoidal dose-response curves showing a gradual increase in activity that plateaued at higher concentrations (Fig. 2h). Half-maximal effective concentrations (EC_50_) were calculated *via* non-linear regression analysis of these curves to quantify the relative potency of each compound. The results illustrated that EC_50_ values were 20.5 μg/mL for CG and 18.8 μg/mL for DG. As expected, the positive control Poly I:C demonstrated the strongest immunostimulatory potency with an EC_50_ of 8.1 μg/mL, which could activate multiple innate immune receptors such as TLR3, RIG-I and MDA5, thereby triggering extensive innate immune responses. These results define the dose-dependent activation profiles of the compounds and identify CG and DG as the potent OAS agonists that activate NF-κB signaling among the tested benzothiazole derivatives (Fig. 4e-g).

Based on the results of knockout and enzymatic activity assays confirming that CG and DG activate the OAS-mediated innate immune signaling pathway, we firstly evaluated the binding affinity of CG and DG to OAS and their interaction with OAS *via* surface plasmon resonance (SPR) assays. Both CG and DG exhibited strong binding affinity to OAS protein, with dissociation constants (Kd) of 4.2 μM and 6.8 μM, respectively, which revealed slightly stronger binding of CG to OAS than DG to OAS (Fig. 4h-i and Fig. S21).

We further performed molecular docking analysis using AutoDock Vina to investigate their direct interaction with human OAS (PDB ID: 4IG8). Molecular docking simulations provide a preliminary structural basis for the structure-activity relationship (SAR) between CG and DG. While both compounds stably occupy the conserved ligand-binding pocket of OAS with comparable predicted binding affinities (DG: −6.518 kcal/mol; CG: −6.328 kcal/mol), the C6 substituent difference likely modulates local interaction patterns. Specifically, the hydroxyl group (-OH) in CG acts as both a hydrogen bond donor and acceptor, forming stable polar interactions with Lys 213 and Glu 233 in the OAS pocket and reinforcing the local hydrogen-bond network (Fig. 4j-k and Tables S2–S3). In contrast, the methoxy group (-OMe) in DG lacks hydrogen bond donor capacity due to methylation, resulting in limited local hydrogen-bonding interactions, which is consistent with the SPR results, indicating that may partially contribute to the differential biological activities observed between the two compounds. Collectively, the integration of molecular docking and SPR analyses provides a robust structural and mechanistic framework for the SAR between CG and DG. Therefore, in this study, we developed non-nucleotide small molecules targeting OAS-2-5A-RIG-I pathway to initiate innate immune responses.

### 2.5 CG and DG enhance protective humoral and cellular immunity against RSV

**Fig. 5:**
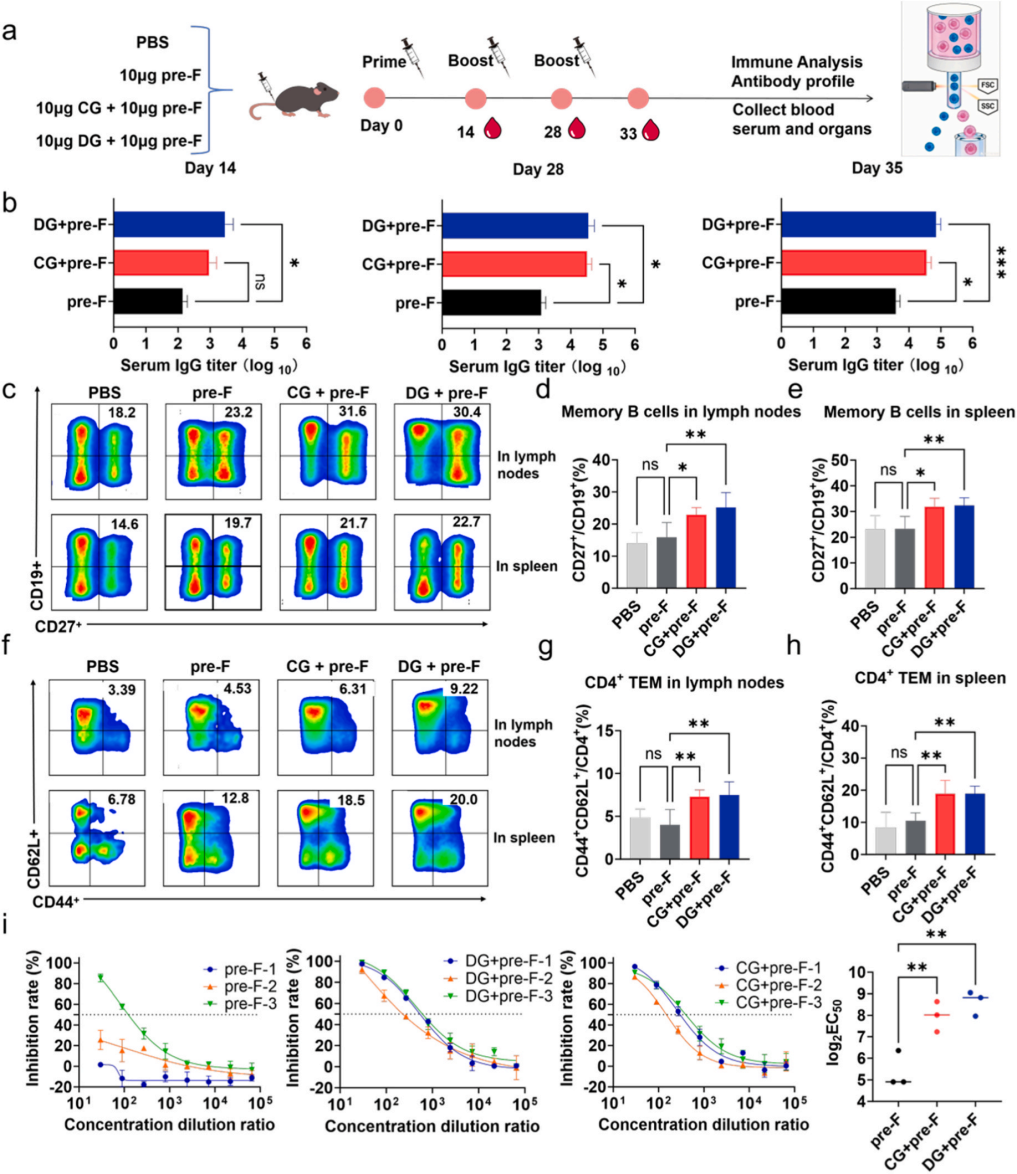
**CG and DG safely enhance memory immune responses and high titers of RSV neutralizing antibodies.** (a) Schematic diagram of the immunization program for C57BL/6J mice: primary immunization on day 0, booster immunizations on days 14 and 28, and blood samples collected on days 14, 28, and 35. (b) ELISA detection of RSV-specific IgG antibody titers in mouse serum on days 14, 28, and 35. Representative flow cytometry scatter plots (c) and quantitative analysis of CD19^+^CD27^+^ memory B cells in the lymph nodes (d) and spleen (e). Representative flow cytometry scatter plots (f) and quantitative analysis of CD4^+^CD44^+^CD62L^+^ T cells in the lymph nodes (g) and spleen (h). Data are represented as mean ± SD (n = 5). (i) RSV neutralizing antibody titers (NT50) in mouse serum on day 35. Data are represented as mean ± SD (n = 3); one-way ANOVA was used for statistical analysis. *p < 0.05, **p < 0.01, ***p < 0.001, ****p < 0.0001, ns indicates no statistically significant difference.

Building upon the elucidation of the immune activation mechanisms of CG and DG, we further evaluated the practical efficacy and safety of benzothiazole compounds CG and DG as adjuvants in RSV vaccine systems. Considering the crucial function of RSV F protein in viral invasion and its conformation-dependent antigenic characteristics, this study selected the highly protective prefusion F protein (pre-F) as the model antigen [35]. C57BL/6J mice were immunized subcutaneously with either the pre-F protein alone or in combination with CG or DG on day 0, followed by booster immunizations on days 14 and 28 (Fig. 5a).

The intensity of humoral immune responses was dynamically assessed by collecting peripheral serum at different time points and detecting RSV-specific IgG antibody titers. The results showed that on day 14 after the initial immunization, compared with the pre-F antigen alone, the CG + pre-F and DG + pre-F groups induced approximately 6.6-fold and 20.6-fold increases in antibody titers, respectively (Fig. 5b). With increasing immunization cycles, antibody titers in all groups showed an increasing trend, but the CG + pre-F and DG + pre-F groups consistently maintained a significant advantage. By day 35, the antibody titers in the CG + pre-F and DG + pre-F groups had reached 9.3 times and 18.7 times that of the antigen-only groups, respectively (Fig. 5b), demonstrating the powerful amplifying effect of adjuvants on the strength and durability of the immune response. The IgG2c/IgG1 ratio is an indicator of Th1/Th2 cell polarization. On day 35, CG and DG groups induced a higher IgG2c/IgG1 ratio than pre-F group treatments, suggesting that CG and DG promoted a Th1-biased immune response (Fig. S22).

Given that long-term protection relies on immune memory, we analyzed memory immune cell populations in the spleen and lymph nodes after the last immunization. Flow cytometry results showed that in the lymph nodes, the proportions of CD19^+^CD27^+^ memory B cells in the CG + pre-F and DG + pre-F groups were 1.43-fold and 1.59-fold higher than those in the pre-F group, respectively (Fig. 5c and d). This enhancement was even more pronounced in the spleen, with the proportions of memory B cells in the CG + pre-F and DG + pre-F groups reaching 1.86-fold and 1.92-fold higher than those in the pre-F group, respectively (Fig. 5c and e). Besides humoral immunity, effective RSV defense also relies on cellular immunity, especially the antigen-specific T-cell responses and memory T cell population capable of clearing infected host cells. The flow cytometry results show that, compared with antigen alone, co-administration with CG or DG significantly increases the frequencies of IFN-γ^+^ and TNF-α^+^ CD4^+^ and CD8^+^ T cells. Flow cytometry and quantitative analyses indicate that both adjuvants effectively enhance antigen-specific Th1-polarized CD4^+^ T cell responses and promote functional CD8^+^ T cell responses (Figure S23 and 24). Further analysis of CD4^+^ T cell subsets revealed that in the lymph nodes, the proportions of CD4^+^CD44^+^CD62L^+^ central memory T cells (TCM) in the CG + pre-F and DG + pre-F groups were 1.37-fold and 1.39-fold higher, respectively, than in the pre-F group (Fig. 5f and g). In the spleen, these proportions were further increased, reaching 1.81-fold and 1.83-fold higher, respectively, than in the pre-F group (Fig. 5f and h). These data indicate that CG and DG not only enhance the initial antibody response but also synergistically establish robust B-cell and T-cell immune memory, supporting the establishment of a comprehensive and durable *anti*-RSV immune barrier.

The goal of protective immunization is to generate neutralizing antibodies capable of blocking viral infection. To verify the protective efficacy of the antibodies, we used a Reed-Muench neutralization assay to detect RSV neutralizing antibody levels in serum. The neutralizing antibody titer was defined as the serum dilution resulting in 50% inhibition of viral infection (NT50). We found that the titer in the pre-F antigen alone group was relatively low (1:30). However, the titers in the CG + pre-F group and the DG + pre-F group were as high as 1:256 and 1:320, respectively, an increase of approximately one order of magnitude (Fig. 5i). This indicates that CG and DG can effectively enhance the vaccine-induced virus neutralization capacity, suggesting enhanced potential for protection against RSV infection.

While the immune enhancement effect is significant, the biosafety of the novel adjuvant is equally crucial. Therefore, we comprehensively tested serum biochemical markers of liver function in mice after immunization. Biochemical analysis showed that compared with the PBS control group, there were no significant differences in the levels of alanine aminotransferase (ALT), aspartate aminotransferase (AST), and alkaline phosphatase (ALP) between the CG + pre-F and DG + pre-F groups (Fig. S25). Meanwhile, at day 35, histopathological examination of major organs was performed. H&E staining of key organs including the heart, liver, spleen, lung, and kidney revealed generally normal tissue architecture across all treatment groups (PBS, pre-F alone, CG + pre-F, and DG + pre-F), with no significant inflammation, necrosis, or structural abnormalities detected (Fig. S26).

Adjuvant-induced local inflammatory reactions typically manifest within 24-72 h post-injection, most commonly as acute erythema, edema, and other local inflammatory signs. Thus, at 72 h after immunization, macroscopic assessment of injection site reactogenicity was performed. Visual examination of the injection site revealed no visible signs of erythema, edema, or tissue necrosis across all treatment groups (PBS, pre-F alone, CG + pre-F, and DG + pre-F), indicating the absence of significant local inflammatory reactions (Fig. S27). This indicates that, at effective immunization doses, these two benzothiazole derivative adjuvants did not induce detectable hepatotoxicity, supporting favorable safety profiles.

To further rule out lysosomal membrane permeabilization (LMP) caused by cationic chemical structures, we have supplemented dedicated LMP detection experiments. RAW264.7 cells were treated with CG and DG at 25 μM for 4 h, and LysoTracker Red staining combined with flow cytometry and confocal laser scanning microscopy was applied to evaluate lysosomal integrity. The results showed that the fluorescence intensity of LysoTracker Red in CG/DG treatment groups was comparable to the blank control group, and no disappearance of punctate lysosomal fluorescence was observed. These data verify that CG and DG do not induce lysosomal membrane permeabilization under experimental conditions (Fig. S28).

In summary, CG and DG enhanced RSV pre-F vaccine immunogenicity by promoting high-titer antigen-specific and neutralizing antibody responses together with durable B- and T-cell memory, while maintaining favorable biocompatibility, highlighting their potential as chemically defined OAS-targeting vaccine adjuvants.

## 3 Discussion

Adjuvants based on PAMPs play critical roles in subunit/recombinant vaccines. These PAMPs are recognized by PRRs to elicit innate immune responses. Toll-like receptors on cellular surfaces or endosomal membranes include 10 subfamilies in humans [36]. PAMPs such as Poly I:C, MPLA, CpG ODN, and imiquimod are potent agonists of different TLRs and have been used as adjuvants in subunit vaccines and clinical trials [[37], [38], [39]]. PAMPs are also recognized by cytosolic receptors, including NOD-like receptors (NLRs), DNA sensors, and RIG-I-like receptors (RLRs) [40]. Cytosolic dsDNA from pathogens or damaged cells activates cGAS, leading to 2′,3′-cGAMP production and STING activation [37,41]. Consequently, 2′,3′-cGAMP acts as a STING agonist for enhancing immune responses against infection and tumors, but is limited by poor stability caused by hydrolysis of phosphate ester bonds, motivating development of non-nucleotide agonists such as MSA-2 and SR-717 [23,42].

As a key RLR, RIG-I senses viral RNA and induces type I interferon production [13]. In this process, OAS, an interferon-stimulated gene encoding dsRNA sensors, is activated by viral dsRNA and generates 2-5A, which activates RNase L to degrade dsRNA in coordination with RIG-I signaling.^43^It is noteworthy that RIG-I-like receptors mainly respond to viral dsRNAs and OAS activation promotes RNase L-mediated degradation of viral RNAs. The OAS–RIG-I axis is therefore central to antiviral immunity. However, 2-5A is unstable and hydrophilic, limiting cellular delivery. Although poly I:C can activate OAS, it suffers from heterogeneity, poor reproducibility, structural instability, and short *in vivo* half-life. In contrast, non-nucleotide small molecules with defined structures enable scalable production, stable quality, and improved storage [[43], [44], [45]]. Therefore, such molecules are urgently needed as OAS–RIG-I axis agonists for vaccine adjuvant development.

This study reports benzothiazole derivatives as small-molecule vaccine adjuvants. Initial SAR analysis was limited to substitutions at the N3 and C6 positions, while further optimization at other positions (C4, C5, C7) is ongoing. Experimental data demonstrate that these compounds activate RIG-I signaling by targeting OAS, leading to type I interferon induction and dendritic cell maturation. Transcriptomic analysis revealed upregulation of OAS family genes in THP-1 cells treated with CG and DG, and gene set enrichment analysis further confirmed activation of interferon-related pathways. CRISPR/Cas9-mediated OAS knockout abolished CG- and DG-induced upregulation of RIG-I and IFN-β, confirming OAS dependence. In vitro assays further showed that CG and DG directly activate OAS in a concentration-dependent manner. Mechanistically, OAS activation triggers RNase L–mediated RNA cleavage, generating immunostimulatory RNA fragments that can amplify RIG-I signaling and downstream type I interferon responses, thereby enhancing antigen-presenting cell maturation and adaptive immune activation. Collectively, these findings establish an OAS–RIG-I–based adjuvant strategy distinct from canonical TLR- and STING-targeting approaches [46,47].

Given that the OAS–RNase L pathway is associated with RNA degradation and potential apoptosis under strong stimulation [48,49], we evaluated the cytotoxicity of CG and DG in antigen-presenting cells. Cell viability remained above 90% at concentrations ≤80 μg/mL, and the effective dose for immune activation (50 μg/mL) was within this non-cytotoxic range (Fig. S29). These results suggest that CG and DG can activate immune signaling without inducing overt cytotoxicity, consistent with a controlled activation of the pathway rather than global RNA degradation.

In terms of chemical structure, a series of benzothiazole derivatives were obtained by introducing different substituents at the R1 and R2 positions. G-series compounds bearing a benzyl group at the N3 position generally exhibited higher immune activation than E-series analogues with an ethyl substitution. Representative compounds BE, CE, CG, and DG were selected for EC_50_ evaluation in RAW-BLUE cells. The measured EC_50_ values were 18.8 μg/mL for DG, 20.5 μg/mL for CG, 22.7 μg/mL for BE and 35.1 μg/mL for CE (Fig. 4g-i and Fig. S30). Collectively, G-series compounds showed lower EC50 values than E-series counterparts, suggesting that increased hydrophobic substitution may favor biological activity, potentially through altered target interactions.

Substituent variation at the R1 position, such as hydroxyl or methoxy groups, further influenced signaling outcomes. CG preferentially induced interferon-related gene expression, whereas DG showed a bias toward NF-κB-related responses, reflecting tunable immune signaling profiles within the benzothiazole scaffold. Consistently, SPR and molecular docking analyses supported differential binding to OAS, with CG showing stronger experimental binding affinity than DG and distinct predicted interaction modes within the OAS pocket. These differences likely contribute to their divergent downstream signaling behaviors, suggesting that subtle structural modifications can tune both binding strength and immune response bias.

In the RSV pre-F protein vaccine model, CG and DG significantly enhanced antigen-specific immune responses, increasing RSV-specific IgG titers and neutralizing antibody levels, while also promoting memory B- and T-cell responses. Importantly, these compounds demonstrated robust *in vivo* immunostimulatory activity while maintaining a favorable biocompatibility profile at effective doses, with no biochemical evidence of hepatotoxicity, supporting their potential as OAS-targeting vaccine adjuvants and the feasibility of chemically defined modulation of the OAS–RIG-I axis. Within the scope of this study, their immunostimulatory effects are more consistent with OAS-dependent pathway engagement rather than nonspecific cellular stress, although more detailed evaluation of stress-related pathways, including ER stress and oxidative stress, will be required to further delineate long-term safety and off-target effects. Further optimization of the benzothiazole scaffold is warranted to refine potency and immune bias.

In summary, this study identified two benzothiazole derivatives, CG and DG, that target OAS. These non-nucleoside small molecules were demonstrated to activate type I interferon signaling and promote dendritic cell maturation *via* the OAS-RIG-I axis, thereby exhibiting effective adjuvant activity in RSV subunit vaccines. This study not only expands the application of the benzothiazole backbone in immunomodulation but also presents unique molecular adjuvants targeting key nodes of innate immunity. The ability of CG and DG to enhance both humoral and cellular immunity highlights their potential as chemically defined adjuvant candidates for vaccines against viral infections.

## 4 Methods

### 4.1 Mice

Pathogen-free female C57BL/6J mice (5-8 weeks old) were obtained from the Laboratory Animal Center of Sun Yat-sen University (SYSU-IACUC-2023-000951). All experimental procedures were approved by the Institutional Animal Care and Use Committee (IACUC) of Sun Yat-sen University and performed in accordance with institutionally approved animal care and use protocols and guidelines. All mice were housed at the Laboratory Animal Center of Sun Yat-sen University under the approved protocols.

### 4.2 Materials and reagents

Phosphate-buffered saline (PBS) was purchased from Gibco (Invitrogen, USA). Horseradish peroxidase (HRP)-conjugated anti-mouse IgG antibody was obtained from Abcam (Cambridge, UK). Anti-mouse CD16/CD32 antibody, fluorophore-labeled anti-mouse CD11c, CD80, CD86 antibodies, Zombie Violet™ Fixable Viability Kit, and anti-mouse CD3, CD4, CD44, CD62L, CD19, CD27, CD45, IFN-γ^+^ and TNF-α^+^ antibodies were purchased from BioLegend (San Diego, USA). PrimeScript™ RT Master Mix, TRIzol Reagent, and SYBR Premix Ex Taq (2×) were obtained from TaKaRa (Beijing, China). BCA protein assay kit was purchased from YEASEN (China). TMB Two-Component Substrate solution (for ELISA) was purchased from Beijing Solarbio Science & Technology Co., Ltd (Beijing, China). Mouse Lymphocyte Isolation Solution was purchased from Dakewe (Shenzhen, China). RSV pre-F A2 was provided by SINO Biological (Beijing, China). Protease inhibitor and Radio-Immunoprecipitation Assay Lysis Buffer (RIPA lysis buffer) were purchased from Beyotime (Shanghai, China). Rabbit *anti*-TBK1 (phospho-Ser 172) antibody, Rabbit *anti*-TBK1 antibody, Rabbit anti-RIG-I antibody were purchased from Abcam (Cambridge, UK). Rabbit *anti*-GAPDH antibody was obtained from Affinity Biosciences (Hangzhou, China). Sterile cell culture dishes and sterile centrifuge tubes were purchased from Guangzhou Jet Bio-Filtration Co., Ltd. (Guangzhou, China). RNase L-IN-1, RIG012 were purchased from MCE (Shanghai, China).

### 4.3 Synthesis of benzothiazole derivatives

Unless otherwise specified, all reagents and solvents were purchased from commercial sources and used without further purification. Under a nitrogen atmosphere, benzothiazole (6.7 mmol) and ethyl iodide (or benzyl bromide, 20.1 mmol) were added to a 250 mL round-bottomed flask containing 70 mL anhydrous acetonitrile. The mixture was heated at 95 °C for 20 h, then cooled to room temperature. The solvent was removed under reduced pressure to afford a white solid. Upon addition of diethyl ether (20 mL), a white precipitate formed, which was isolated by filtration, thoroughly washed with diethyl ether, and dried in vacuo. ^1^HNMR were recorded on AVANCE NEO Ascend 400 at room temperature. Chemical shifts are referenced to the residual solvent peak and reported in ppm (*δ* scale), and all coupling constant (J) values are given in Hz. HRMS data was measured on MAT95XP. The purity of all compounds used in biological assays was confirmed by elemental analysis, the elemental composition of each compound deviated by less than 0.4%.

#### 4.3.1 3-Ethyl-2-methylbenzo[d]thiazol-3-ium iodide (AE)

Yield: 48%. White solid. MP: 193.0 - 196.4 °C. ^1^H NMR (400 MHz, DMSO) *δ* 8.47 (d, *J* = 7.7 Hz, 1H), 8.35 (d, *J* = 8.4 Hz, 1H), 7.93 - 7.86 (m, 1H), 7.80 (t, *J* = 7.3 Hz, 1H), 4.78 (q, *J* = 7.3 Hz, 2H), 3.23 (s, 3H), 1.46 (t, *J* = 7.3 Hz, 3H).

#### 4.3.2 3-Benzyl-2-methylbenzo[d]thiazol-3-ium bromide (AG)

Yield: 90%. Pink solid. MP: 207.1 - 211.0 °C. 1H NMR (400 MHz, DMSO) *δ* 8.52 (d, J = 7.8 Hz, 1H), 8.21 (d, J = 8.3 Hz, 1H), 7.91 - 7.73 (m, 2H), 7.46 - 7.28 (m, 5H), 6.11 (s, 2H), 3.28 (s, 3H), 2.51 (s, 3H).

#### 4.3.3 3-Ethyl-2,6-dimethylbenzo[d]thiazol-3-ium iodide (BE)

Yield: 50%. White solid. MP: 163.5 - 170.2 °C. ^1^H NMR (400 MHz, MeOD) *δ* 8.15 (d, *J* = 8.8 Hz, 1H), 8.10 (s, 1H), 7.75 (dd, *J* = 8.8, 1.9 Hz, 1H), 4.79 (t, *J* = 7.4 Hz, 2H), 2.59 (s, 3H), 1.58 (t, *J* = 7.4 Hz, 3H).

#### 4.3.4 3-Benzyl-2,6-dimethylbenzo[d]thiazol-3-ium bromide (BG)

Yield: 88%. Purple solid. MP: 208.8 - 209.2 °C. ^1^H NMR (400 MHz, CH_3_OH) *δ* 7.93 - 7.54 (m, 2H), 7.28 (dd, *J* = 8.3, 1.7 Hz, 1H), 2.78 (s, 3H), 2.45 (s, 3H).

#### 4.3.5 3-Ethyl-6-hydroxy-2-methylbenzo[d]thiazol-3-ium iodide (CE)

Yield: 75%. White solid. MP: 254.3 - 259.1 °C. ^1^H NMR (400 MHz, DMSO) *δ* 10.60 (s, 1H), 8.13 (d, *J* = 9.2 Hz, 1H), 7.73 (s, 1H), 7.27 (d, *J* = 11.7 Hz, 1H), 4.67 (q, *J* = 7.3 Hz, 2H), 3.12 (s, 3H), 1.43 (t, *J* = 7.3 Hz, 3H).

#### 4.3.6 3-Benzyl-6-hydroxy-2-methylbenzo[d]thiazol-3-ium bromide (CG)

Yield: 75%. White solid. MP: 249.5 - 255.7 °C. ^1^H NMR (400 MHz, DMSO) *δ* 10.64 (s, 1H), 8.02 (d, *J* = 9.2 Hz, 1H), 7.80 (d, *J* = 2.4 Hz, 1H), 7.44 - 7.17 (m, 6H), 6.02 (s, 2H), 3.18 (s, 3H).

#### 4.3.7 3-Ethyl-6-methoxy-2-methylbenzo[d]thiazol-3-ium bromide (DE)

Yield: 45%. White solid. MP: 174.3 - 174.9 °C. ^1^H NMR (400 MHz, DMSO) *δ* 8.24 (d, *J* = 9.3 Hz, 1H), 8.03 (s, 1H), 7.47 (d, *J* = 9.3 Hz, 1H), 4.72 (q, *J* = 7.2 Hz, 2H), 3.90 (s, 3H), 3.16 (s, 3H), 1.44 (t, *J* = 7.3 Hz, 3H).

#### 4.3.8 3-Benzyl-6-methoxy-2-methylbenzo[d]thiazol-3-ium bromide (DG)

Yield: 85%. White solid. MP: 195.6 - 204.1 °C. ^1^H NMR (400 MHz, DMSO) *δ* 8.10 (d, *J* = 9.3 Hz, 1H), 8.07 (d, *J* = 2.9 Hz, 1H), 7.49 - 7.17 (m, 6H), 6.05 (s, 2H), 3.88 (s, 3H), 3.21 (s, 3H).

### 4.4 Cell culture

RAW264.7 cells (mouse macrophage cell line) were used for preliminary screening of compound cytotoxicity and inflammatory cytokine secretion profiles. HEK293 cells were used to prepare retroviral or lentiviral particles required for gene knockdown or overexpression experiments. HEK293 and RAW 264.7 cell lines were maintained in complete Dulbecco's Modified Eagle Medium (cDMEM) supplemented with 10% fetal bovine serum (FBS) and 1% penicillin-streptomycin. THP-1 cells (human monocyte cell line) were used for signaling pathway analysis. The isolation and culture methods for mouse bone marrow-derived dendritic cells (BMDCs) are detailed in the Methods section below and were used for maturity determination. THP-1 cells and BMDCs were cultured in complete RPMI 1640 medium (cRPMI) containing 10% heat-inactivated FBS and 1% penicillin-streptomycin. In the following experiment THP-1 cells were treated with PBS, AE, AG, BE, BG, CE, CG, DE, DG and 2′,3′-cGAMP of 50 μg/mL.

### 4.5 Lentiviral production, concentration, and generation of knockout cell lines

HEK293 cells were seeded in 6-well plates at a density of 2 × 10^5^ cells/well and cultured overnight until 70-80% confluency. Transfection was performed using Lipofectamine 2000 (Thermo Fisher Scientific, USA) following the manufacturer's instructions: briefly, 3 μg of recombinant plasmid and 9 μL of Lipofectamine 2000 were separately diluted in Opti-MEM reduced-serum medium (Thermo Fisher Scientific, USA), incubated at room temperature for 5 min, then mixed and incubated for another 20 min to form transfection complexes. The complexes were added to the cell culture medium, and the cells were cultured at 37 °C with 5% CO_2_. At 6 h post-transfection, the medium was replaced with fresh complete DMEM medium. Cell culture supernatants were harvested at 48 h post-transfection, and then cell-free supernatants were clarified at 3000 × g for 15 min, filtered through 0.22 μm membranes and subjected to ultracentrifugation at 100,000 × g for 2 h at 4 °C (Type 70 Ti rotor, Beckman Coulter). The resulting viral pellets resuspended in PBS with 1% FBS were added in THP-1 cells. Mix gently and incubate the virus with THP-1 cells at room temperature for 20 min under a tissue-culture hood then centrifuge at 800 × g for 30 min. Remove the supernatant (virus-containing medium) and resuspend the cell pellet in 3 mL fresh cRPMI. Cells were allowed to expand before analyzing stable knockout efficiency of OAS or IFI16 by qPCR. In subsequent experiments, KO-OAS and KO-IFI16 cells were treated with CG or DG (50 μg/mL).

The gene-specific crRNA sgRNA sequences targeting OAS or IFI16 were cloned: For OAS KO cells we used the gene (1#:5′-GACTGAGTTCGCTCCAGCTCG-3′), (2#:5′-GTCATCCGCCTAGTCAAGCAC-3′) or (3#:5′-GAGGCCGATCTGACGCTGACC-3′); for IFI16 KO cells we used the gene (1#:5′-GTATACCAACGCTTGAAGACC-3′), (2#:5′-GGACCAGCCCTATCAAGAAAG-3′) or (3#:5′-GAAAACGCCCAGTGATAGTGA-3′).

### 4.6 Western blotting

Cells were seeded in 6-well plates at a density of 2 × 10^6^ cells per well and cultured overnight. The old medium was replaced with fresh medium. After 24 h of incubation, cells were harvested and lysed in RIPA buffer supplemented with 1 mM PMSF and 1× phosphatase inhibitor cocktail. Lysis was accelerated using an ultrasonic cell disruptor. Cell lysates were centrifuged at 12,000×g for 20 min at 4 °C. The supernatants were collected, and protein concentrations were determined using a bicinchoninic acid (BCA) kit. Equal amounts of protein (40 μg) were mixed with SDS-PAGE loading buffer and heated at 95 °C for 10 min. Proteins were separated by 10% SDS-PAGE and transferred to 0.45 μm polyvinylidene difluoride (PVDF) membranes. Membranes were blocked with 5% bovine serum albumin (BSA) in PBS for 2 h at 37 °C, then incubated with primary antibodies [TBK1 (1:1000), p-TBK1 (1:1000), and GAPDH (1:4000)] at 4 °C overnight. After incubation, membranes were washed five times with PBS containing 0.05% Tween 20 (PBST) and then incubated with HRP-conjugated goat anti-rabbit secondary antibody (1:10,000) at room temperature for 1 h. Membranes were washed five times with PBST and visualized using an ImageQuant LAS 500 imaging system (GE Healthcare, USA).

### 4.7 BMDC activation assay

Female C57BL/6J mice (6-8 weeks old) were purchased from the Laboratory Animal Center of Sun Yat-sen University. All animal experiments were conducted under protocols approved by the IACUC of Sun Yat-sen University (approval number: SYSU-IACUC-2023-000951). Bone marrow-derived dendritic cells (BMDCs) were isolated and cultured as previously described. Briefly, BMDCs were extracted from C57BL/6J mice and cultured in complete RPMI 1640 medium supplemented with 20 ng/mL granulocyte-macrophage colony-stimulating factor (GM-CSF) and 10 ng/mL interleukin-4 (IL-4) for 6 days. After differentiation, BMDCs were harvested, seeded in 48-well plates at a density of 2 × 10^5^ cells/well, and cultured overnight. The old medium was replaced with fresh medium containing PBS, AE, AG, BE, BG, CE, CG, DE, DG, or 2′,3′-cGAMP (final concentration: 50 μg/mL). After 24 h of incubation, cells were collected and resuspended in PBS containing 1% BSA for flow cytometric analysis. For staining, cells were blocked with anti-mouse CD16/CD32 antibody for 30 min. After two washes with PBS, fluorophore-labeled anti-mouse CD11c, anti-mouse CD80, and anti-mouse CD86 antibodies were added, and cells were incubated in the dark for 30 min. Cells were washed twice with PBS and resuspended in PBS containing 1% BSA for flow cytometry.

### 4.8 Quantitative reverse transcription PCR (qRT-PCR)

qRT-PCR was performed to detect relative expression in THP-1 cells or KO cells. Cells were treated as indicated, then washed with PBS. Total RNA was extracted using TRIzol Reagent according to the manufacturer's instructions. Complementary DNA (cDNA) was synthesized from total RNA using PrimeScript™ RT Master Mix. qRT-PCR was performed using SYBR Premix Ex *Taq*II (Tli RNaseH Plus) with specific primers (Integrated DNA Technologies) on an Applied Biosystems StepOnePlus Real-Time PCR System. The thermal cycling conditions were as follows: initial denaturation at 95 °C for 30 s; 45 cycles of denaturation at 95 °C for 5 s and annealing/extension at 60 °C for 30 s; and a final melting curve analysis at 95 °C for 15 s, 60 °C for 1 min, and 95 °C for 15 s.

Primer sequences are listed.

GAPDH: 5′-ACATCATCCCTGCCTCTACTG-3′,

IFI16: 5′-TTGATTAGAAGTGCCAGCGTA-3′,

RIG-I: 5′-GTGGAATCACGGATTAGC-3′,

OAS: 5′-GAAGGAAAGGTGCTTCCGAGGTAG-3′

IFN-β:5′-GCTTGGATTCCTACAAAGAAGCA-3’.

### 4.9 ELISA for cytokine quantification

Cytokine concentrations were measured using enzyme-linked immunosorbent assay (ELISA) kits according to the manufacturer's protocols. THP-1 cells were treated as indicated for 24 h. Cell culture supernatants were collected to determine tumor necrosis factor-α (TNF-α) concentrations using Human TNF-alpha ELISA Kit (Abcam, USA). Absorbance was measured at 450 nm using a Synergy 2 microplate reader (BioTek, USA), and cytokine concentrations were calculated based on the standard curves provided in each kit.

### 4.10 RNA-seq analysis

Total RNA was extracted from PBS, CG, DG treated THP-1 cells using TRIzol Reagent. Library preparation was performed by Gene Denovo (Guangzhou, China): mRNA was enriched with oligo(dT) beads, fragmented, and reverse-transcribed into cDNA. After end-repair, adaptor ligation, and size selection (∼200 bp), cDNA was PCR-amplified and purified. Sequencing was done on an Illumina platform, generating paired-end reads. Clean reads were aligned to the Ensembl release 110 with HISAT2. Transcript reconstruction and expression quantification were performed using StringTie and RSEM. Differentially expressed genes were identified with DESeq2.

### 4.11 Detection of humoral and cellular immune responses

Mice (6-8 weeks old, *n* = 5) were immunized subcutaneously with PBS, 10 μg pre-F, 10 μg CG + 10 μg pre-F, or 10 μg DG + 10 μg pre-F on day 0. Mice received booster immunizations with the same doses on day 14 and day 28 post-primary vaccination. Mice immunized with PBS or pre-F alone served as controls. Blood samples were collected on day 35 after primary immunization, and serum was separated to determine IgG titers.

Cellular immune responses to different formulations were evaluated in the same mice used for IgG titer detection. After splenic red blood cell lysis, cells were blocked with anti-mouse CD16/32 at 4 °C for 30 min. Following centrifugation at 1500 rpm for 10 min, Zombie violet viability dye (0.5 μL per 100 μL sample) was added and incubated for 30 min. Cells were washed three times with flow staining buffer, then stained with anti-mouse CD3, CD45, CD4 and CD8 antibodies at 4 °C for 30 min to label surface markers. RSV pre-F-specific memory T cells and memory B cells in the spleen and lymph nodes were analyzed by flow cytometry. Cells were stimulated with RSV F peptide pools in the presence of brefeldin A before intracellular cytokine staining. After repeated washes, cells were fixed and permeabilized with Fixation Buffer at room temperature for 20 min, then rinsed twice with Wash buffer. Cells were incubated with fluorophore-conjugated anti-mouse IFN-γ and TNF-α antibodies on ice for 30 min for intracellular staining. Post staining, cells were washed twice with Wash buffer and analyzed on an Attune NXT Acoustic Focusing Cytometer (Life Technology, USA). Data quantification was performed using FlowJo software (TreeStar, USA).

### 4.12 Determination of antigen-specific IgG titers

96-well ELISA plates (Jet Bio-Filtration Co., Ltd., China) were coated overnight in carbonate-bicarbonate buffer (pH 9.6) with 1 μg/mL OVA or 1 μg/mL RSV pre-F protein and blocked at 37 °C for 2 h with 3% BSA in PBS. Serum samples were serially diluted with 1% BSA in PBS and added to 96-well plates pre-coated with OVA or RSV pre-F protein. After incubation at 37 °C for 2 h, the plates were thoroughly washed with PBST (PBS + 0.05% Tween-20), and HRP-labeled anti-mouse IgG (Abcam, USA, diluted 1:50,000) was added and incubated at 37 °C for 1 h. After washing, the plates were developed with TMB substrate (Solarbio, China) and the reaction was terminated with 2 M H_2_SO_4_. The absorbance was measured at 450 nm using a Synergy 2 microplate reader (BioTek, USA). The endpoint titer was defined as the reciprocal of the highest dilution that produced an OD_450_ value greater than 2.1 times the mean background value of the negative control wells.

### 4.13 RSV neutralization assay

RSV neutralization activity was evaluated using a Reed–Muench assay. Briefly, serially diluted mouse sera were incubated with RSV A2 strain at 37 °C for 1 h and subsequently added to HEp-2 cells. After incubation for 24 h, viral infection was assessed by immunostaining or cytopathic effect analysis. The 50% neutralization endpoint was calculated using the Reed–Muench method.

### 4.14 Determination of EC_50_ for NF-κB activation in RAW-Blue cells

RAW-Blue™ cells (InvivoGen, USA) were seeded and treated with BE, CE, CG, or DG (5, 10, 20, 40, 80, 160, 320, 640 μg/mL), or the positive control Poly I:C (1, 2.5, 5, 10, 20, 40, 80, 160, μg/mL) for 24 h. Supernatants were collected and incubated with QUANTI-Blue™ Solution (InvivoGen, catalog code: rep-qbs), at 37 °C. Absorbance at 620 nm was measured using a Synergy 2 microplate reader (BioTek, USA). EC_50_ values were calculated *via* non-linear regression based on dose-response curves.

### 4.15 Molecular docking

Molecular docking was performed to predict the binding mode of CG or DG to 2-5A synthetase 1 (OAS). The crystal structure of human OAS (PDB ID: 4IG8) was obtained from the RCSB Protein Data Bank (RCSB PDB). Protein pretreatment was performed using AutoDock Vina v1.2.7. Crystal water molecules and redundant components with non-specific binding were removed, polar hydrogen atoms were added to simulate the protonated state under physiological conditions, Gasteiger charges were assigned, and non-polar hydrogens were merged. The processed protein was saved as a PDBQT file for subsequent docking. The three-dimensional (3D) structure of the target compound CG and DG were constructed using Chem3D software and optimized for energy minimization using the MMFF94 force field to obtain a low-energy stable conformation. Subsequently, AutoDock Tools was used to detect and define rotatable bonds, assign Gasteiger charges, and save the structure as a PDBQT file for subsequent docking. A docking grid box was constructed around the known active pocket of OAS, with its center and dimensions determined based on the spatial distribution of key residues in the active site to ensure full coverage of the potential binding region. Molecular docking was performed using AutoDock Vina v1.2.7 with default parameters. Each ligand was configured to output a maximum of nine binding modes, which were then sorted according to predicted binding affinity (kcal/mol). The root-mean-square deviation (RMSD) of each binding mode relative to the top-ranked pose was calculated, including both lower bound (RMSD l.b.) and upper bound (RMSD u. b.) values, to assess structural differences between conformations.

### 4.16 Surface plasmon resonance analysis

CM5 chips were used for immobilization of OAS protein. Buffers matching protein isoelectric point were adopted to improve immobilization efficiency, followed by ethanolamine blocking and reference channel setting. CG and DG were dissolved in DMSO and diluted with running buffer with consistent DMSO proportion to eliminate solvent interference. Gradient samples were repeatedly injected to record full-time sensor grams. After reference subtraction, blank correction and baseline alignment, relevant response data were exported. Global 1:1 kinetic fitting and Langmuir affinity fitting were performed, with baseline drift, residual error and repeatability strictly detected for quality control.

### 4.17 Hematoxylin and eosin (H&E) staining

Tissue sections were deparaffinized in xylene twice (5 min each) and hydrated through a graded ethanol series (100%, 95%, 80%, 70%, 3 min each), followed by rinsing with distilled water for 2 min. Sections were stained with hematoxylin for 5 min, differentiated in hydrochloric acid-alcohol for 30 s, and blued in running water for 5 min. After eosin staining for 30 s, sections were dehydrated in a graded ethanol series (70%, 80%, 95%, 100%, 3 min each), cleared in xylene twice (5 min each), and mounted with neutral balsam. The stained sections were scanned and analyzed using a TissueFA XS SpectraS multispectral tissue imaging analysis system (TissueGnostics GmbH, Austria).

### 4.18 LMP detection experiments

RAW264.7 macrophages were seeded in 20 mm confocal dishes at a density of 1.5 × 10^4^ cells per dish with 1 mL complete medium and incubated with CG or DG for 4 h. After removing culture medium, cells were washed three times with PBS. For lysosome labeling, LysoTracker Red was diluted 100-fold in medium (10 μL dye mixed with 1 mL medium), and 200 μL diluted stain was added to each dish followed by incubation at 37 °C for 30 min. Cells were then washed with PBS three times, fixed with 4% paraformaldehyde for 15-20 min, and rinsed with PBS again. Subsequently, nuclei were stained with Hoechst 33,342, which was prepared with the same 100-fold dilution protocol as LysoTracker Red; 200 μL diluted Hoechst solution was applied to each dish and incubated at 37 °C for 10 min. After final PBS washing, cellular co-localization signals were captured under a laser scanning confocal microscope.

### 4.19 Statistical analysis

Statistical analyses were performed using one-way analysis of variance (ANOVA) with GraphPad Prism software (version 7.0; GraphPad Software, USA). P values < 0.05 were considered statistically significant.

## Data availability

All data generated or analyzed in this study are included in this published article (and its supplementary information files).

## Notes

The authors declare no competing financial interest.

## Ethics approval and consent to participate

Pathogen-free female C57BL/6J mice (5-8 weeks old) were obtained from the Laboratory Animal Center of Sun Yat-sen University (SYSU-IACUC-2023-000951). All experimental procedures were approved by the Institutional Animal Care and Use Committee (IACUC) of Sun Yat-sen University and performed in accordance with institutionally approved animal care and use protocols and guidelines. All mice were housed at the Laboratory Animal Center of Sun Yat-sen University under the approved protocols.

## CRediT authorship contribution statement

**Xuanli Jin:** Conceptualization, Data curation, Formal analysis, Investigation, Methodology, Visualization, Writing – original draft. **Zhihui Zhang:** Methodology, Validation. **Hong Liu:** Formal analysis, Methodology. **Yunzhi Tan:** Methodology. **Peiying Luo:** Methodology. **Zhenfu Wen:** Conceptualization, Investigation, Methodology, Validation, Writing – original draft. **Haolin Chen:** Conceptualization, Formal analysis, Investigation, Methodology, Validation, Writing – original draft, Writing – review & editing. **Lixin Liu:** Conceptualization, Funding acquisition, Project administration, Resources, Supervision, Writing – original draft, Writing – review & editing. **Yongming Chen:** Conceptualization, Funding acquisition, Project administration, Resources, Supervision, Writing – original draft, Writing – review & editing.

## Declaration of competing interest

The authors declare that they have no known competing financial interests or personal relationships that could have appeared to influence the work reported in this paper.
