# Supplementary material for: Benzothiazole derivatives target oligoadenylate synthetase and enhance RSV vaccine efficacy as adjuvants
Source: Bioact Mater. 2026 Aug 4;67:80–94. doi: 10.1016/j.bioactmat.2026.07.049 (PMC13463801; doi:10.1016/j.bioactmat.2026.07.049)
Supplement: Supplementary file 1 [file mmc1.docx]

**Supporting Information**

**Benzothiazole Derivatives Target** **Oligoadenylate Synthetase and Enhance RSV Vaccine Efficacy as Adjuvants**

**Table of Content:**

S. No. Pages

**Supplementary Figures**	**S3**

**Supplementary Tables**	**S32**

# **Supplementary Figures**

**Supplementary Figure S1.** The ^1^H NMR spectrum of AE in DMSO‑d_6_.

**Supplementary Figure S2.** The ^1^H NMR spectrum of BE in DMSO‑d_6_.

**Supplementary Figure S3.** The ^1^H NMR spectrum of CE in DMSO‑d_6_.

**Supplementary Figure S4.** The ^1^H NMR spectrum of DE in DMSO‑d_6_.

**Supplementary Figure S5.** The ^1^H NMR spectrum of AG in DMSO‑d_6_.

**Supplementary Figure S6.** The ^1^H NMR spectrum of BG in DMSO‑d_6_.

**Supplementary Figure S7.** The ^1^H NMR spectrum of CG in DMSO‑d₆.

**Supplementary Figure S8.** The ^1^H NMR spectrum of DG in DMSO‑d₆.

Elemental Composition Report

Single Mass Analysis

Tolerance = 10.0 PPM / DBE: min = -1.5, max = 80.0

Elements Used:

C: 0-30 H: 0-60 N: 0-5 O: 0-5 S: 1-1

| Mass | Calc. Mass | mDa | PPM | DBE | Formula |
| --- | --- | --- | --- | --- | --- |
| 178.0692 | 178.069 | 0.2 | 1.1 | 5.5 | C_10_H_12_NS |

**Supplementary Figure S9.** The high-resolution mass spectrum of AE.

Elemental Composition Report

Single Mass Analysis

Tolerance = 10.0 PPM / DBE: min = -1.5, max = 80.0

Elements Used:

C: 0-30 H: 0-60 N: 0-5 O: 0-5 S: 1-1

| Mass | Calc. Mass | mDa | PPM | DBE | Formula |
| --- | --- | --- | --- | --- | --- |
| 192.0849 | 192.0847 | 0.2 | 1 | 5.5 | C_11_H_14_NS |

**Supplementary Figure S10.** The high-resolution mass spectrum of BE.

Elemental Composition Report

Single Mass Analysis

Tolerance = 10.0 PPM / DBE: min = -1.5, max = 80.0

Elements Used:

C: 0-30 H: 0-60 N: 0-5 O: 0-5 S: 1-1

| Mass | Calc. Mass | mDa | PPM | DBE | Formula |
| --- | --- | --- | --- | --- | --- |
| 194.0636 | 194.064 | -0.4 | -2.1 | 5.5 | C_10_H_13_NOS |

**Supplementary Figure S11.** The high-resolution mass spectrum of CE.

Elemental Composition Report

Single Mass Analysis

Tolerance = 10.0 PPM / DBE: min = -1.5, max = 80.0

Elements Used:

C: 0-30 H: 0-60 N: 0-5 O: 0-5 S: 1-1

| Mass | Calc. Mass | mDa | PPM | DBE | Formula |
| --- | --- | --- | --- | --- | --- |
| 208.0794 | 208.0796 | -0.2 | -1 | 5.5 | C_11_ H_15_NOS |

**Supplementary Figure S12.** The high-resolution mass spectrum of DE.

Elemental Composition Report

Single Mass Analysis

Tolerance = 10.0 PPM / DBE: min = -1.5, max = 80.0

Elements Used:

C: 0-30 H: 0-60 N: 0-5 S: 1-1

| Mass | Calc. Mass | mDa | PPM | DBE | Formula |
| --- | --- | --- | --- | --- | --- |
| 240.0852 | 240.0847 | 0.5 | 2.1 | 9.5 | C_15_H_14_NS |

**Supplementary Figure S13.** The high-resolution mass spectrum of AG.

Elemental Composition Report

Single Mass Analysis

Tolerance = 10.0 PPM / DBE: min = -1.5, max = 80.0

Elements Used:

C: 0-30 H: 0-60 N: 0-5 S: 1-1

| Mass | Calc. Mass | mDa | PPM | DBE | Formula |
| --- | --- | --- | --- | --- | --- |
| 254.1002 | 254.1003 | -0.1 | -0.4 | 9.5 | C_16_H_17_NS |

**Supplementary Figure S14.** The high-resolution mass spectrum of BG.

**Supplementary Figure S15.** Western blot analysis of relative RIG-I signals in THP-1 cell treated with PBS, 50 μg/mL of positive control poly I:C, or 50 μg/mL of benzothiazole.

**Supplementary Figure S16.** RT-qPCR analysis of relative IFN-β mRNA expression levels in THP-1 cell treated with PBS, 50 μg/mL of CG, DG, CM, DM for 24 h (PBS group as control).

**Supplementary Figure S17.** Gating strategy for CD80/CD86 expression on CD11c^+^ BMDCs.

**Supplementary** **Figure S18.** Basal mRNA expression level of OAS in KO-IFI16 THP-1 cells (a) and KO-OAS THP-1 cells (b) compared to WT cells. Cells with lower mRNA expression were selected for the experiment in the main text.

**Supplementary** **Figure S19.** (a) Western blot analysis of relative p-TBK1 signal in wild-type (WT), IFI16-knockout (KO-IFI16), and OAS-knockout (KO-OAS) cells treated with PBS, CG, or DG, (b) normalized to total TBK1 to evaluate the dependence of TBK1 phosphorylation on the OAS pathway.

**Supplementary** **Figure S20.** Relative IFN-β mRNA expression in G/DG-treated THP-1 cells with or without RNase L/RIG-I inhibitors.

**Supplementary** **Figure S21.** Global kinetic fitting and residual distribution of OAS–CG and OAS–DG. The lower panel displays the residual plot (in Response Units, RU).

**Supplementary** **Figure S22.** Serum IgG2c/IgG1 ratio on day 35 were measured in mice vaccinated with different formulation with ELISA.

**Supplementary Figure S23.** CG and DG adjuvants markedly boost RSV pre-F-specific functional CD4^+^ and CD8^+^ T cell responses. (a, c) Representative flow cytometry dot plots of IFN-γ and TNF-α production by splenic CD4^+^ T cells from immunized mice. Numbers in gated boxes indicate the frequencies of antigen-specific cytokine-positive cells. (b, d) Quantitative bar graphs summarizing the percentages of CD4^+^IFN-γ^+^ and CD4^+^TNF-α^+^ T cells. (e, g) Representative flow cytometry dot plots of IFN-γ and TNF-α production by splenic CD8^+^ T cells from immunized mice. Numbers in gated boxes indicate the frequencies of antigen-specific cytokine-positive cells. (f, h) Quantitative bar graphs summarizing the percentages of CD8^+^IFN-γ^+^ and CD8^+^TNF-α^+^ T cells. Data are presented as mean ± SD. **p* < 0.05, ***p* < 0.01.

**Supplementary Figure S24.** Gating strategy for intracellular cytokine staining of antigen-specific functional CD4⁺ and CD8⁺ T cells. The sequential contour plots at the top illustrate the hierarchical gating workflow for lymphocyte and T cell identification. First, total lymphocytes were gated based on FSC-A and SSC-A parameters; single cells were further isolated to exclude doublets. Total T lymphocytes were defined as CD45⁺CD3⁺ populations, followed by segregation into CD4⁺ helper T cells and CD8⁺ cytotoxic T cells using CD4 and CD8 fluorescent signals. The four contour plots at the bottom show representative staining profiles for antigen-specific CD8^+^IFN-γ^+^, CD8^+^TNF-α^+^, CD4^+^IFN-γ^+^, and CD4^+^TNF-α^+^T cell subsets.

**Supplementary** **Figure S25.** Detection of liver function indicators (ALT, AST, ALP) in mouse serum on day 35. Data are represented as mean ± SD (n=3).

**Supplementary** **Figure S26.** Representative photomicrographs of H&E-stained tissue sections from major organs (heart, liver, spleen, lung, and kidney) collected from mice.

**Supplementary** **Figure S27.** Representative images of local reactogenicity at the subcutaneous injection site in mice 72 hours after vaccination.

**Supplementary** **Figure S28. Immunofluorescence staining of RAW 264.7 cells treated with CG or DG.** Lysosomes were labeled with LysoTracker Red (red channel), and cell nuclei were counterstained with Hoechst 33342 (blue channel). WT: untreated control cells; CG: CG-treated group; DG: DG-treated group. Merged images show the colocalization of lysosomal and nuclear signals.

**Supplementary** **Figure S29.** Cell viability was measured by CCK-8 assay after 24 h incubation with CG and DG at graded concentrations (0, 10, 20, 40, 80, 160 μg/mL). Data are shown as mean ± SD, n = 3.

**Supplementary** **Figure S30.** Dose-response profiles of benzothiazole derivatives in RAW-BLUE cells treated with BE, CE (5, 10, 20, 40, 80, 160, 320, 640 μg/mL) for 24 h, with NF-κB activation measured via SEAP activity and EC₅₀ values determined by non-linear regression.

# **Supplementary Tables**

**Supplementary Table S1.** Comparison of Measured and Theoretical Elemental Composition (wt%)

**Supplementary Table S2. Molecular docking results of CG binding to OAS protein**

| **Mode** | **Affinity**  **(kcal/mol)** | **Dist from best mode** | |
| --- | --- | --- | --- |
|  |  | **RMSD l.b.** | **RMSD u.b.** |
| 1 | -6.328 | 0 | 0 |
| 2 | -6.163 | 13.46 | 15 |
| 3 | -6.158 | 13.41 | 15.17 |

Mode: Ranked binding pose number sorted by docking affinity (1 = most favorable pose). Affinity: Predicted binding affinity calculated by AutoDock Vina, expressed in kcal/mol. Lower (more negative) values indicate stronger binding. RMSD l.b.(RMSD lower bound) and RMSD u.b. (RMSD upper bound): Root-mean-square deviation (RMSD, in Å) of each pose relative to the best pose (mode 1). RMSD l.b. is calculated by matching each heavy atom to its closest counterpart in the reference pose; RMSD u.b. is calculated by one-to-one atom matching.

**Supplementary Table S3.** **Molecular docking results of DG binding to OAS protein**

| **Mode** | **Affinity**  **(kcal/mol)** | **Dist from best mode** | |
| --- | --- | --- | --- |
|  |  | **RMSD l.b.** | **RMSD u.b.** |
| 1 | -6.518 | 0 | 0 |
| 2 | -6.146 | 4.231 | 6.284 |
| 3 | -6.135 | 1.997 | 2.93 |

Mode: Ranked binding pose number sorted by docking affinity (1 = most favorable pose). Affinity: Predicted binding affinity calculated by AutoDock Vina, expressed in kcal/mol. Lower (more negative) values indicate stronger binding. RMSD l.b.(RMSD lower bound) and RMSD u.b. (RMSD upper bound): Root-mean-square deviation (RMSD, in Å) of each pose relative to the best pose (mode 1). RMSD l.b. is calculated by matching each heavy atom to its closest counterpart in the reference pose; RMSD u.b. is calculated by one-to-one atom matching.
